# Supplementary material for: PICU Survivorship: Factors Affecting Feasibility and Cohort Retention in a Long-Term Outcomes Study
Source: Children (Basel). 2022 Jul 13;9(7):1041. doi: 10.3390/children9071041 (PMC9317147; doi:10.3390/children9071041)
Supplement: Supplementary file 1 [file children-09-01041-s001.zip › Table S1.pdf]

POP Admission Survey

How many years old is your child today?

(If your child has not yet reached his/her first birthday, enter 0). ______________________________

Parent completing survey
- ◯ Mother
- ◯ Father
- ◯ Other

If other, please specify ______________________________

In the following questions, we will be asking about your child's overall health and development.

First, we would like to ask you about your child's health.

Is there a place your child usually goes when he/she is sick or you need advice about his/her health?
- ◯ Yes
- ◯ No

What kind of place does your child go to when he/she is sick or you need advice about his/her health?
- ◯ Doctor's office
- ◯ Hospital emergency room
- ◯ Hospital outpatient department
- ◯ Clinic or health center
- ◯ School (nurse's office, athletic trainer's office, etc.)
- ◯ Friend/relative
- ◯ Mexico/other locations out of US
- ◯ Some other place
- ◯ Does not go to one place most often
- ◯ Don't know

Is there a place that your child usually goes when he/she needs routine preventative care, such as a physical exam or a well-child check up?
- ◯ Yes
- ◯ No

What kind of place does your child go for routine preventative care?
- ◯ Doctor's office
- ◯ Hospital emergency room
- ◯ Hospital outpatient department
- ◯ Clinic or health center
- ◯ School (nurse's office, athletic trainer's office, etc.)
- ◯ Friend/relative
- ◯ Mexico/other locations out of US
- ◯ Some other place
- ◯ Does not go to one place most often
- ◯ Don't know

Do you have one or more persons you think of as your child's personal doctor or nurse?
- ◯ Yes
- ◯ No

Who is this person? ______________________________

During the past 12 months, how many times did your child receive a well-child check-up, that is a general check-up, when he/she was not sick or injured?	________________________________
During the past 12 months, how many times did your child visit a hospital emergency room?	________________________________
During the past 12 months, how many times was your child admitted to the hospital (not including current admission)?	________________________________
During the past 12 months, was your child admitted to the intensive care unit?	◯ Yes<br>◯ No
Has your child ever seen any of the following specialists? (Check all that apply.)	☐ Cardiologist<br>☐ Dermatologist<br>☐ Developmental/Behavioral Pediatrician<br>☐ Endocrinologist<br>☐ Gastroenterologist<br>☐ Hematologist<br>☐ Hepatologist<br>☐ Infectious Disease Specialist<br>☐ Nephrologist<br>☐ Neurologist<br>☐ Neurosurgeon<br>☐ Oncologist<br>☐ Orthopedic Surgeon<br>☐ Otolaryngologist (ENT)<br>☐ Pediatric/Trauma Surgeon<br>☐ Physical Medicine and Rehabilitation Physician/Physiatrist<br>☐ Plastic Surgeon<br>☐ Psychiatrist<br>☐ Pulmonologist<br>☐ Rheumatologist<br>☐ Sleep Medicine Specialist<br>☐ Transplant Surgeon<br>☐ Other
If other, please specify:	________________________________
When was the last time your child saw a cardiologist?	◯ 0-1 month ago<br>◯ 2-3 months ago<br>◯ 4-6 months ago<br>◯ 7-9 months ago<br>◯ 10-12 months ago<br>◯ Longer than 12 months ago
When was the last time your child saw a dermatologist?	◯ 0-1 month ago<br>◯ 2-3 months ago<br>◯ 4-6 months ago<br>◯ 7-9 months ago<br>◯ 10-12 months ago<br>◯ Longer than 12 months ago

When was the last time your child saw a developmental/behavioral pediatrician?	◯ 0-1 month ago<br>◯ 2-3 months ago<br>◯ 4-6 months ago<br>◯ 7-9 months ago<br>◯ 10-12 months ago<br>◯ Longer than 12 months ago
When was the last time that your child saw an endocrinologist?	◯ 0-1 month ago<br>◯ 2-3 months ago<br>◯ 4-6 months ago<br>◯ 7-9 months ago<br>◯ 10-12 months ago<br>◯ Longer than 12 months ago
When was the last time your child saw a gastroenterologist?	◯ 0-1 month ago<br>◯ 2-3 months ago<br>◯ 4-6 months ago<br>◯ 7-9 months ago<br>◯ 10-12 months ago<br>◯ Longer than 12 months ago
When was the last time your child saw a hematologist?	◯ 0-1 month ago<br>◯ 2-3 months ago<br>◯ 4-6 months ago<br>◯ 7-9 months ago<br>◯ 10-12 months ago<br>◯ Longer than 12 months ago
When was the last time your child saw a hepatologist?	◯ 0-1 month ago<br>◯ 2-3 months ago<br>◯ 4-6 months ago<br>◯ 7-9 months ago<br>◯ 10-12 months ago<br>◯ Longer than 12 months ago
When was the last time your child saw an infectious disease specialist?	◯ 0-1 month ago<br>◯ 2-3 months ago<br>◯ 4-6 months ago<br>◯ 7-9 months ago<br>◯ 10-12 months ago<br>◯ Longer than 12 months ago
When was the last time your child saw a nephrologist?	◯ 0-1 month ago<br>◯ 2-3 months ago<br>◯ 4-6 months ago<br>◯ 7-9 months ago<br>◯ 10-12 months ago<br>◯ Longer than 12 months ago
When was the last time your child saw a neurologist?	◯ 0-1 month ago<br>◯ 2-3 months ago<br>◯ 4-6 months ago<br>◯ 7-9 months ago<br>◯ 10-12 months ago<br>◯ Longer than 12 months ago

When was the last time your child saw a neurosurgeon?	◯ 0-1 month ago<br>◯ 2-3 months ago<br>◯ 4-6 months ago<br>◯ 7-9 months ago<br>◯ 10-12 months ago<br>◯ Longer than 12 months ago
When was the last time your child saw an oncologist?	◯ 0-1 month ago<br>◯ 2-3 months ago<br>◯ 4-6 months ago<br>◯ 7-9 months ago<br>◯ 10-12 months ago<br>◯ Longer than 12 months ago
When was the last time your child saw an otolaryngologist?	◯ 0-1 month ago<br>◯ 2-3 months ago<br>◯ 4-6 months ago<br>◯ 7-9 months ago<br>◯ 10-12 months ago<br>◯ Longer than 12 months ago
When was the last time your child saw a pediatric/trauma surgeon?	◯ 0-1 month ago<br>◯ 2-3 months ago<br>◯ 4-6 months ago<br>◯ 7-9 months ago<br>◯ 10-12 months ago<br>◯ Longer than 12 months ago
When was the last time your child saw a physical medicine and rehabilitation physician?	◯ 0-1 month ago<br>◯ 2-3 months ago<br>◯ 4-6 months ago<br>◯ 7-9 months ago<br>◯ 10-12 months ago<br>◯ Longer than 12 months ago
When was the last time your child saw a psychiatrist?	◯ 0-1 month ago<br>◯ 2-3 months ago<br>◯ 4-6 months ago<br>◯ 7-9 months ago<br>◯ 10-12 months ago<br>◯ Longer than 12 months ago
When was the last time your child saw a pulmonologist?	◯ 0-1 month ago<br>◯ 2-3 months ago<br>◯ 4-6 months ago<br>◯ 7-9 months ago<br>◯ 10-12 months ago<br>◯ Longer than 12 months ago
When was the last time your child saw a rheumatologist?	◯ 0-1 month ago<br>◯ 2-3 months ago<br>◯ 4-6 months ago<br>◯ 7-9 months ago<br>◯ 10-12 months ago<br>◯ Longer than 12 months ago

When was the last time your child saw a sleep medicine specialist?	◯ 0-1 month ago<br>◯ 2-3 months ago<br>◯ 4-6 months ago<br>◯ 7-9 months ago<br>◯ 10-12 months ago<br>◯ Longer than 12 months ago
When was the last time your child saw an orthopedic surgeon?	◯ 0-1 month ago<br>◯ 2-3 months ago<br>◯ 4-6 months ago<br>◯ 7-9 months ago<br>◯ 10-12 months ago<br>◯ Longer than 12 months ago
When was the last time your child saw a plastic surgeon?	◯ 0-1 month ago<br>◯ 2-3 months ago<br>◯ 4-6 months ago<br>◯ 7-9 months ago<br>◯ 10-12 months ago<br>◯ Longer than 12 months ago
When was the last time your child saw a transplant surgeon?	◯ 0-1 month ago<br>◯ 2-3 months ago<br>◯ 4-6 months ago<br>◯ 7-9 months ago<br>◯ 10-12 months ago<br>◯ Longer than 12 months ago
If "other" was marked, when was the last time your child saw this other physician?	◯ 0-1 month ago<br>◯ 2-3 months ago<br>◯ 4-6 months ago<br>◯ 7-9 months ago<br>◯ 10-12 months ago<br>◯ Longer than 12 months ago
Has your child ever received any of the following therapies? (Check all that apply.)	☐ Behavioral therapy<br>☐ Feeding therapy<br>☐ Occupational therapy<br>☐ Physical therapy<br>☐ Speech therapy<br>☐ Talk therapy (with a social worker, psychologist, or psychiatrist)<br>☐ Other
If other, please specify:	__________________________________
When was the last time your child received behavioral therapy?	◯ 0-1 month ago<br>◯ 2-3 months ago<br>◯ 4-6 months ago<br>◯ 7-9 months ago<br>◯ 10-12 months ago<br>◯ Longer than 12 months ago
When was the last time your child received feeding therapy?	◯ 0-1 month ago<br>◯ 2-3 months ago<br>◯ 4-6 months ago<br>◯ 7-9 months ago<br>◯ 10-12 months ago<br>◯ Longer than 12 months ago

Question	Response
When was the last time your child received occupational therapy?	◯ 0-1 month ago<br>◯ 2-3 months ago<br>◯ 4-6 months ago<br>◯ 7-9 months ago<br>◯ 10-12 months ago<br>◯ Longer than 12 months ago
When was the last time your child received physical therapy?	◯ 0-1 month ago<br>◯ 2-3 months ago<br>◯ 4-6 months ago<br>◯ 7-9 months ago<br>◯ 10-12 months ago<br>◯ Longer than 12 months ago
When was the last time your child received speech therapy?	◯ 0-1 month ago<br>◯ 2-3 months ago<br>◯ 4-6 months ago<br>◯ 7-9 months ago<br>◯ 10-12 months ago<br>◯ Longer than 12 months ago
When was the last time your child received talk therapy?	◯ 0-1 month ago<br>◯ 2-3 months ago<br>◯ 4-6 months ago<br>◯ 7-9 months ago<br>◯ 10-12 months ago<br>◯ Longer than 12 months ago
Does your child receive home nursing care?	◯ Yes<br>◯ No
How many hours/week does your child receive home nursing care?	______________________
Has a doctor or other health care provider ever told you that your child has any of the following conditions?	☐ Anxiety problems<br>☐ Attention Deficit Disorder or Attention Deficit Hyperactive Disorder (ADD or ADHD)<br>☐ Autism or other autism spectrum disorder<br>☐ Behavioral or conduct problems<br>☐ Depression<br>☐ Developmental delay<br>☐ Epilepsy or seizure disorder<br>☐ Intellectual disability or mental retardation<br>☐ Learning disorder<br>☐ Other
If other, please specify:	______________________
Does your child take medications for behavior, attention, or mood problems?	◯ Yes<br>◯ No
Which medications?	______________________

Next, we will ask questions about how your child is doing at school.

Does your child attend school outside of your home?	◯ Yes<br>◯ No
What grade is your child in school?	◯ Kindergarten<br>◯ 1st Grade<br>◯ 2nd Grade<br>◯ 3rd Grade<br>◯ 4th Grade<br>◯ 5th Grade<br>◯ 6th Grade<br>◯ 7th Grade<br>◯ 8th Grade<br>◯ 9th Grade<br>◯ Other
If other, please specify:	__________________________________
Is your child in a Special Education Program?	◯ Yes<br>◯ No
Does your child have an IEP (Individualized Educational Program)?	◯ Yes<br>◯ No
What services and accommodations does your child receive in his/her IEP? (Choose all that apply.)	☐ Extended school year<br>☐ Feeding Therapy<br>☐ Occupational Therapy<br>☐ One-on-one aide<br>☐ Physical Therapy<br>☐ Social Skills Group<br>☐ Social Work<br>☐ Speech Therapy<br>☐ Other
Was your child in Early Intervention (before the age of 3 years)?	◯ Yes<br>◯ No
What services did your child receive through Early Intervention? (Check all that apply.)	☐ Developmental Therapy<br>☐ Feeding Therapy<br>☐ Nutritional Therapy<br>☐ Occupational Therapy<br>☐ Physical Therapy<br>☐ Social Work<br>☐ Speech Therapy<br>☐ Other
If other, please specify:	__________________________________
Over the past 12 months, how many school days did your child miss because of illness or injury?	__________________________________

Next, we will ask you about additional impacts that your child's health may have had.

How many other children live in your household? __________________________________

In the past 12 months, on average, how many school days did each of these children miss due to your child's illness?

Child 1 __________________________________

Child 2 __________________________________

Child 3 __________________________________

Child 4 __________________________________

Child 5 __________________________________

Child 6 __________________________________

Child 7 __________________________________

Has your child's health conditions caused financial problems for your family?
- ◯ Yes
- ◯ No

Have you or other family members stopped working because of your child's health conditions?
- ◯ Yes
- ◯ No

Have you or other family members cut down on the hours you work because of your child's health?
- ◯ Yes
- ◯ No
